# L'arbre qui cache la forêt

**DOI:** 10.11604/pamj.2015.22.84.7778

**Published:** 2015-10-01

**Authors:** Naziha Khammassi, Youssef Kort

**Affiliations:** 1 Service de Médecine Interne, Hôpital Razi 2010, La Manouba, Tunisie, Faculté de médecine de Tunis, Tunisie

**Keywords:** Complément, atteinte cutanée, lupus de l′enfant, Complement, skin involvement, child lupus

## Image en medicine

Le lupus est une pathologie chronique, qui évolue par poussées. Son expression clinique est très polymorphe et chaque symptôme peut être inaugural. Il peut débuter dès l'enfance, ces formes sont rares et de pronostic sévère. Le déficit en fractions du complément constitue l'un des principaux facteurs génétiques prédisposant au lupus érythémateux systémique. Nous rapportons un cas de lupus érythémateux chronique avec déficit en fraction C2 du complément révélé par une atteinte cutanée. Patiente âgée de 10 ans était suivie en dermatologie pour des lésions cutanées, apparues un an auparavant, à type d’éruption érythémato-papuleuse touchant le nez et les joues, des placards érythémateux finement squameux des mains et des pieds. Il existait, par ailleurs, une chéilite érosive et une plaque squameuse alopécique du cuir chevelu dont l'examen direct était négatif. Plusieurs diagnostics étaient évoqués au début (eczéma, toxidermies, photodermatose…). L'histologie était en faveur d'un lupus érythémateux chronique en poussée et l'immunofluorescence directe montrait une faible fluorescence micro granuleuse IgG, IgM et C3 à la jonction dermo-épidermique. Le bilan immunologique initial avait objectivé des anticorps antinucléaires faiblement positifs à 1/160, des anticorps anti-DNA négatifs et des anti-SSA positifs. Le dosage du complément montrait un déficit homozygote en fraction C2 du complément: C2 fonctionnel < 10% (normale 70 à 115%) avec des taux normaux de C3 et C4. Le bilan de systématisation était négatif. La patiente était traitée par hydroxychloroquine (Plaquénil^®^) à la dose de 4 mg/kg/j et photoprotection externe avec évolution clinique favorable.

**Figure 1:**
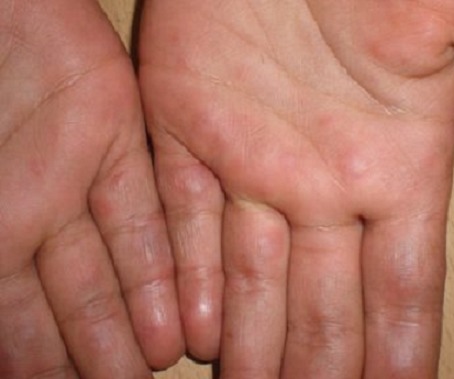
Placards érythémateux finement squameux des mains

